# Transcriptome architecture of the three main lineages of agrobacteria

**DOI:** 10.1128/msystems.00333-23

**Published:** 2023-07-21

**Authors:** Lucas Waldburger, Mitchell G. Thompson, Alexandra J. Weisberg, Namil Lee, Jeff H. Chang, Jay D. Keasling, Patrick M. Shih

**Affiliations:** 1 Department of Bioengineering, University of California, Berkeley, California, USA; 2 Joint BioEnergy Institute, Emeryville, California, USA; 3 Biological Systems and Engineering Division, Lawrence Berkeley National Laboratory, Berkeley, California, USA; 4 Environmental Genomics and Systems Biology Division, Lawrence Berkeley National Laboratory, Berkeley, California, USA; 5 Department of Botany and Plant Pathology, Oregon State University, Corvallis, Oregon, USA; 6 Department of Chemical and Biomolecular Engineering, University of California, Berkeley, California, USA; 7 Institute for Quantitative Biosciences, University of California, Berkeley, California, USA; 8 Novo Nordisk Foundation Center for Biosustainability, Technical University of Denmark, Kongens Lyngby, Denmark; 9 Center for Synthetic Biochemistry, Institute for Synthetic Biology, Shenzhen Institutes for Advanced Technologies, Shenzhen, China; 10 Department of Plant and Microbial Biology, University of California, Berkeley, California, USA; Tufts University, Medford, Massachusetts, USA

**Keywords:** agrobacterium, transcription start sites, differential RNA-seq, evolution, pathogenesis, comparative transcriptomics

## Abstract

Agrobacteria are a diverse, polyphyletic group of prokaryotes with multipartite genomes capable of transferring DNA into the genomes of host plants, making them an essential tool in plant biotechnology. Despite their utility in plant transformation, genome-wide transcriptional regulation is not well understood across the three main lineages of agrobacteria. Transcription start sites (TSSs) are a necessary component of gene expression and regulation. In this study, we used differential RNA-seq and a TSS identification algorithm optimized on manually annotated TSS, then validated with existing TSS to identify thousands of TSS with nucleotide resolution for representatives of each lineage. We extend upon the 356 TSSs previously reported in *Agrobacterium fabrum* C58 by identifying 1,916 TSSs. In addition, we completed genomes and phenotyping of *Rhizobium rhizogenes* C16/80 and *Allorhizobium vitis* T60/94, identifying 2,650 and 2,432 TSSs, respectively. Parameter optimization was crucial for an accurate, high-resolution view of genome and transcriptional dynamics, highlighting the importance of algorithm optimization in genome-wide TSS identification and genomics at large. The optimized algorithm reduced the number of TSSs identified internal and antisense to the coding sequence on average by 90.5% and 91.9%, respectively. Comparison of TSS conservation between orthologs of the three lineages revealed differences in cell cycle regulation of *ctrA* as well as divergence of transcriptional regulation of chemotaxis-related genes when grown in conditions that simulate the plant environment. These results provide a framework to elucidate the mechanistic basis and evolution of pathology across the three main lineages of agrobacteria.

**IMPORTANCE:**

Transcription start sites (TSSs) are fundamental for understanding gene expression and regulation. Agrobacteria, a group of prokaryotes with the ability to transfer DNA into the genomes of host plants, are widely used in plant biotechnology. However, the genome-wide transcriptional regulation of agrobacteria is not well understood, especially in less-studied lineages. Differential RNA-seq and an optimized algorithm enabled identification of thousands of TSSs with nucleotide resolution for representatives of each lineage. The results of this study provide a framework for elucidating the mechanistic basis and evolution of pathology across the three main lineages of agrobacteria. The optimized algorithm also highlights the importance of parameter optimization in genome-wide TSS identification and genomics at large.

## INTRODUCTION

The study of agrobacteria has revolutionized plant molecular genetics by enabling genomic modification of plants (1). Their use in plant biotechnology is a function of their unique pathology, whereby pathogenic strains are capable of interkingdom transfer of DNA into plants resulting in crown gall or hairy root disease (2). Disease pathology is determined by the presence of either a conjugative tumor-inducing (Ti) or root-inducing (Ri) plasmid, which contains the majority of genes required for virulence (2). Although hairy root and crown gall are distinct diseases, both are initiated by plant-derived metabolites which begin a signaling cascade that drives expression of virulence genes on the Ti or Ri plasmid resulting in the transfer of DNA into host genomes (2). Expression by the plant host of genes on the transferred DNA (T-DNA) results in production of the plant hormones auxins and cytokinins that cause uncontrolled cellular growth, as well as the biosynthesis of uncommon nutrients called opines, which serve as a nutrient metabolized by agrobacteria.

**Fig 1:**
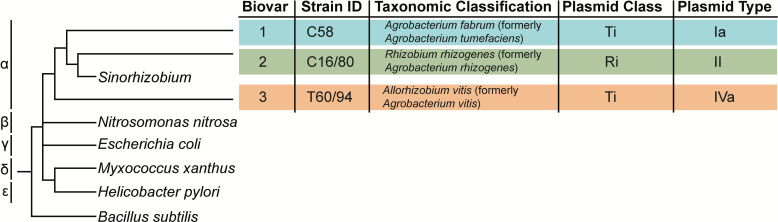
Phylogeny of the three main lineages of agrobacteria. Outgroups for the main groups in Proteobacteria are shown. Within Alphaproteobacteria, representative strains of agrobacteria were selected for each lineage based on their ability to grow in rich and minimal growth media, susceptibility to antibiotics, and oncogenic plasmid. Branch lengths are not to scale. The tip of the tree shows strains selected to represent the three main lineages of agrobacteria, their taxonomic classification, and oncogenic plasmids.

Phylogenetic and genomic analyses indicate that the groups classically referred to as *Agrobacterium*, *Rhizobium*, *Allorhizobium*, *Neorhizobium*, *Ensifer/Sinorhizobium*, *Pararhizobium*, and *Shinella* are related at the genus level (Fig. 1) and are collectively referred to as the agrobacteria-rhizobia complex (ARC) (2, 3). This bacterial clade has been studied for their genetic differences in oncogenic plasmid type leading to distinct phenotypic characteristics, behavior, and pathologies across the ARC (2, 3). The three main lineages of agrobacteria within the ARC are polyphyletic and have traditionally been referred to as biovar (BV) 1, BV2, and BV3 based on physiological and biochemical properties rather than molecular phylogeny (2, 3). BV1, traditionally referred to as *Agrobacterium tumefaciens*, is a species complex that encompasses more than 22 species-level groups called genomospecies (G1–22) (4). It includes *Agrobacterium tumefaciens* (G1) and *Agrobacterium fabrum* (G8)*,* which have traditionally been studied as reference strains with Ti plasmids that lead to crown gall formation in a broad range of plant hosts (1). BV2 has traditionally been referred to as *Agrobacterium rhizogenes*, now *Rhizobium rhizogenes*, is a species-level group of agrobacteria that also have broad host ranges (2). Despite the names, strains of both BV1 and BV2 can carry Ti and/or Ri plasmids as well as cause crown gall or hairy root disease, respectively (4). BV3 is a species complex originally known as *Agrobacterium vitis*, now *Allorhizobium vitis*, whose members can carry Ti plasmids and induce crown gall disease, but its members have been isolated only from grapevine, suggesting a more narrow host range (5, 6).

All strains of agrobacteria sequenced thus far have multipartite genomes (4). Most have a primary circular chromosome and a secondary chromosome that can be either linear or circular (7, 8). Secondary chromosomes, or chromids, have features indicative of being derived from plasmids but also carry essential genes and are, therefore, chromosome-like. Strains may also carry one or many non-oncogenic plasmids that are traditionally referred to as “pAt” plasmids (9). By definition, pathogenic strains carry an oncogenic Ti or Ri plasmid.

Agrobacteria have tunable gene regulation systems to sense and respond to changes in the plant-microbe interface. This is underscored by the complex regulation of *vir* gene expression from the oncogenic plasmid. Multiple environmental signals such as pH, phenolic compounds, and sugars are integrated by the master two-component sensor VirG, which upon phosphorylation activates the expression of the other *vir* genes required for host infection (1, 10). Mutations in *virG* that make it constitutively active are known to result in agrobacterial strains that produce larger and more abundant galls (11), are able to infect a wider range of plants (12), and have significant biotechnological utility (13). Though the expression of *vir* genes and oncogenic plasmid type are critical determinants to host range and disease severity, specific molecular and genetic determinants of these complex pathologies remain unknown—especially within BV2 and BV3 isolates (2). An improved understanding of differences in transcriptional architecture and regulation of these agrobacteria, beyond just *vir* gene regulation, is critical in understanding how these different lineages navigate interactions with plants.

A complete map of microbial transcriptional regulation requires genome-wide information of promoter structure and 5′ untranslated region (UTR) features. A key step in the regulation of bacterial gene expression is the initiation of transcription, where the RNA polymerase binds DNA at the transcriptional start site (TSS) (14). Transcriptional start sites are a primary feature of 5′ UTR and an essential determinant of gene expression. For example, bacteria have been shown to use multiple TSSs depending on stress conditions to enable multiple layers of gene expression control (15). Although the biology of agrobacteria has been well studied in *Sinorhizobium meliloti* and a few reference strains in BV1, the promoter and 5′ UTR structure across their genomes have not been well explored. This understanding is especially limited in BV2 and BV3 where most genome sequences are incomplete. Comparing TSSs on the genomes of these strains will highlight conserved and divergent gene regulation features. Genome-wide TSS identification across the three main lineages will provide a comparative understanding of transcriptional regulation and evolutionary differences in agrobacteria-plant interactions.

Differential RNA sequencing (dRNA-seq) has emerged as a high-throughput method for genome-wide TSS identification (16). This approach utilizes a terminator exonuclease (TEX) to enrich for 5′ triphosphorylated primary transcripts relative to 5′ monophosphated processed transcripts. Comparative analysis between TEX-treated and -untreated samples enables accurate TSS identification based on next-generation sequencing technologies. This method has been used for genome-wide TSS identification in diverse bacterial species, including *Helicobacter pylori* (16), *Escherichia coli* (17), and *Bacillus subtilis* (18). In particular, dRNA-seq has been used to identify TSSs in plant symbionts such as *Bacillus amyloliquefaciens* (18) and *Sinorhizobium meliloti* (19). Biotechnologically relevant bacteria have also been analyzed by dRNA-seq, and the results may be exploited to optimize transcriptional regulation of metabolic pathways (20, 21). For example, the distance of the TSSs relative to RNA polymerase binding sites or the sequence identity of the 5′ UTR has been used to modulate gene expression in synthetic circuits.

Several bioinformatics tools are available for TSS identification from dRNA-seq data (22 - 24), permitting fast and unbiased detection as well as rapid reanalysis after inclusion of new sequencing data. However, these algorithms still require supervision with respect to their sensitivity and specificity. Parameter optimization on manually annotated data is important to minimize false positives, yet this is often overlooked. Previous work provided a comprehensive approach for genome-wide TSS identification by performing manual curation of TSSs and comparing algorithm results to previous manually annotated TSSs across the genome of *Helicobacter pylori* under five different growth conditions (24). Previous work in *Clostridium* species (21) also incorporated optimization by accounting for expression bias from highly expressed genes by incorporating RNA-seq from the same libraries in order to normalize their TSS data set. These results are a reminder that algorithms, especially in genomics, have limited accuracy without performing parameter optimization, then validation on separate subsets of manually annotated data can lead to an inaccurate representation of biological phenomena.

A systematic understanding of transcriptional regulation at a genome scale remains to be elucidated for agrobacteria. We identified genome-wide TSS with nucleotide resolution to understand genome and transcriptional dynamics in the three main lineages of agrobacteria. We found differences in transcriptional regulation of cell cycle and cell wall functions across lineages. We also identified divergent transcriptional regulation during induction conditions that might explain differences in pathologies during plant infection.

## RESULTS

### Characterization of strains representing BV2 and BV3

Complete, finished genome sequences facilitate accurate TSS identification. The type BV1 strain *A. fabrum* C58 was among the first bacteria ever sequenced and has been extensively studied (8); however, for both the BV2 and BV3 clades, there are no representative strains that have been characterized as thoroughly as *A. fabrum* C58. To identify reference strains for the BV2 and BV3 clades, we characterized the growth in defined media of three isolates of both BV2 and BV3 clades that have previously been phylogenetically characterized (Fig. S1) (2). Based on our results, BV2 strain *Rhizobium rhizogenes* C16/80 and BV3 strain *Allorhizobium vitis* T60/94 grew well in rich media, mannitol glutamate yeast extract salts medium (MGYS), and minimal media, morpholinopropanesulfonate (MOPS) based medium; however, neither grew well in Reasoner's 2A (R2A) or lysogeny broth (LB) media (Fig. S1). *R. rhizogenes* C16/80 has an RI type II plasmid and was isolated from *Malus domestica,* while *A. vitis* T60/94 has a Ti type IVa plasmid and was isolated from *Vitis vinifera* (Fig. 1). Both *R. rhizogenes* C16/80 and *A. vitis* T60/94 were tested for antibiotic susceptibility against carbenicillin, gentamicin, kanamycin, chloramphenicol, spectinomycin, tetracycline, hygromycin, and apramycin (Table S1). *R. rhizogenes* C16/80 is susceptible to all tested antibiotics except spectinomycin, whereas *A. vitis* T60/94 is resistant only to carbenicillin. Although both *R. rhizogenes* C16/80 and *A. vitis* T60/94 have previously been sequenced (2), both genome assemblies were drafts, so we used Oxford Nanopore technology for long-read sequencing to close their sequences (Table 1). The genome of *R. rhizogenes* C16/80 is closed and consists of a chromosome, a chromid, two plasmids, and an Ri type II plasmid. The genome of *A. vitis* T60/94 consists of a chromosome, a chromid, two plasmids, and a Ti type IVa plasmid; one of the non-oncogenic plasmids failed to close and is represented by a large contig of 135.7 kb and several small contigs of less than 2 kb. Completion of both *R. rhizogenes* C16/80 and *A. vitis* T60/94 enables comprehensive genome-wide TSS identification of each multipartite genome.

**TABLE 1:** Comparison of agrobacteria genome assemblies^*a*^

Strain	Replicon	Total genes	Size (bp)	GC (%)
*A. fabrum* C58	Circular chromosome	2,749	2,841,580	59.4
	Linear chromosome	1,809	2,075,577	59.3
	At plasmid	476	542,868	57.3
	Ti plasmid	181	214,233	56.7
*R. rhizogenes* C16/80	Chromosome	3,905	4,012,013	60.4
	Chromid	2,614	2,852,423	59.6
	Plasmid 1	159	176,580	58.9
	Plasmid 2 (incomplete)	125	135,788 (largest contig); 143,329 total	56.4
	Ri plasmid	187	203,380	57.1
*A. vitis* T60/94	Chromosome	3,635	3,818,208	57.7
	Chromid	993	1,147,840	57.6
	Plasmid 2	327	315,387	57.7
	At plasmid	578	645,425	56.9
	Ti plasmid	151	157,890	56.4

^*a*^ The total number of annotated genes, size in base pairs, and GC% by replicon across *A. fabrum* C58*, R. rhizogenes* C16/80, and *A. vitis* T60/94. The largest contig was used for R. *rhizogenes* C16/80, the total size of Plasmid 2 is 143,329 bp.

### dRNA-seq reveals the transcriptome architecture of agrobacteria

The study of agrobacteria has largely focused on their unique process of infection, which has led to a detailed understanding of their virulence plasmids. However, the impact of chromosomes and chromids on virulence remains less understood. We aimed to explore the broader transcriptome organization across replicons by identifying TSSs and corresponding promoter structure to inform how agrobacteria virulence has evolved across the three main lineages. Using dRNA-seq data, we identified a total of 6,998 TSSs across the three lineages. Consistent with other Alphaproteobacteria, we observed a conserved promoter structure. Furthermore, we found lineage-specific divergence in TSSs associated with cell cycle regulation, suggesting adaptations in cell cycle control.

Genome-scale TSS identification requires expression from operons across replicons (25). To this end, we isolated RNA from cultures grown in rich media (MGYS) sampled from both logarithmic and stationary phases, which have been shown across multiple bacteria to yield dramatically different transcriptomes (26). Additionally, we isolated RNA from each strain grown in minimal media supplemented with glucose or succinate as a sole carbon source. A minimal medium yields different gene expression patterns than that of bacteria grown in rich media (27). As glucose is metabolized through glycolysis and succinate is metabolized through the TCA cycle, we expected different transcriptional dynamics. Finally, as expression of virulence genes is extremely important to the study of agrobacteria, we isolated RNA from cultures that were resuspended in virulence-inducing buffer (10 mM MES, pH 5.5, 200 µM acetosyringone) for 2 hours (28).

### Optimization of the TSS identification algorithm

**Fig 2:**
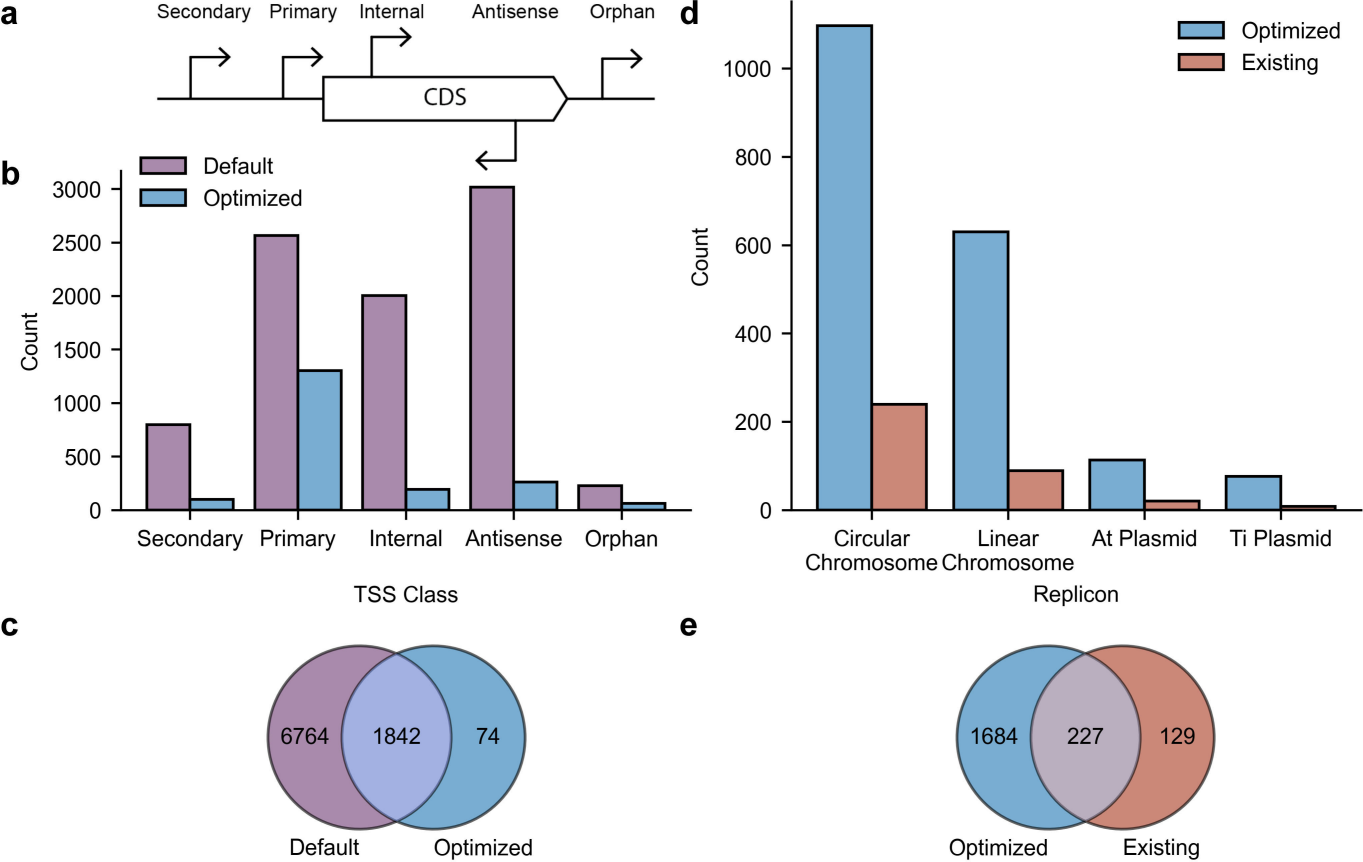
Accurate TSS identification through parameter optimization and validation. (**a**) Classification of TSSs relative to the CDS. (**b**) Overall TSS counts using default and optimized parameters. (**c**) Overlap of TSS counts between the default and optimized data sets. (**d**) Overall TSS counts in the optimized data set and existing data set that were previously reported by Wilms et al. (**e**) Overlap of TSS counts in the optimized and existing data sets.

TSSs are often classified as primary, secondary, antisense, internal, or orphan based on proximity to annotated coding sequence (CDS) (24) as illustrated in Fig. 2a. The primary TSS was defined based on proximity to dRNA-seq reads mapped within 300 bp upstream of the annotated start codon of the CDS. TSSs further upstream of the CDS were classified as secondary TSS. Secondary TSSs are hypothesized to regulate gene expression in specific conditions where polymerases bind to alternative sigma factors (21). Internal TSSs are located inside a CDS on the sense strand. Antisense TSSs are located internal or within 100 bp of a CDS in the antisense strand. A TSS not assigned to any of these categories (i.e., more than 300 bp upstream or more than 100 bp downstream of an annotated CDS) was categorized as an orphan TSS. A TSS can fall into more than one category depending on its location relative to surrounding CDS annotations.

Accurate TSS identification by computational methods requires parameter optimization for improved sensitivity (29). TSS identification with default parameters has been shown to have lower sensitivity resulting in false positives (29). We used ANNOgesic (29) to select optimal parameters on a manually annotated subset of TSSs across each of the replicons of *A. fabrum* C58 with variable read depths. Parameter optimization reduced the number of detected TSSs relative to those predicted when using default parameters, especially for antisense and internal TSS. Examining how parameter optimization affected TSS identification across TSS classes in *A. fabrum* C58 revealed the greatest reduction of TSSs within the antisense (90.4% reduction), internal (91.3% reduction), secondary (87.2% reduction), and orphan (73% reduction) classes, while the primary TSS (45.5% reduction) class was less depleted (Fig. 2b).

We assessed the consistency of the TSS class distribution in *A. fabrum* C58 with that of *R. rhizogenes* C16/80 and *A. vitis* T60/94. In *R. rhizogenes* C16/80, optimization reduced internal TSSs by 91.5% and antisense TSSs by 92.9%. In *A. vitis* T60/94, optimization reduced internal TSSs by 89.6% and antisense TSSs by 91.6% (Fig. S3). Although some antisense and internal TSSs drive expression of truncated proteins or non-coding RNAs (ncRNAs), these transcripts have been observed by other genome-wide TSS studies and have been shown to mainly be the result of transcriptional noise, arising at spurious promoters throughout the genome (30).

In comparison to previous genome-wide TSS identification in *E. coli*, which used this algorithm to identify 5,574 internal and 5,495 antisense TSSs (17), this brings into question the widespread misuse of computational methods resulting from inadequate parameter tuning. Using the default parameters, we identified 8,606 TSSs in *A. fabrum* C58. Parameter optimization reduced the total number of detected TSSs to 1,916 in *A. fabrum* C58. Comparing the overlap of identified TSS, 1,842 optimized TSS are also found in the default parameter data sets in *A. fabrum* C58 (Fig. 2c).

### Validating the performance of the optimized TSS algorithm

Next, we compared the predicted TSSs to those previously reported. Previous efforts at identifying TSSs of sRNA in *A. fabrum* C58 also found 356 primary TSS corresponding to CDS across its replicons (31). We extended these findings by identifying 1,302 primary TSSs, 99 secondary TSSs, 192 internal TSSs, 262 antisense TSSs, and 61 orphan TSSs for *A. fabrum* C58 in the optimized data set (Fig. 2d). Of the 356 primary TSSs previously reported in *A. fabrum* C58, 227 (64.3%) were also found within our data set while also identified an additional 1,687 novel TSSs (Fig. 2e). There are 129 previously reported primary TSSs that we did not identify (Fig. 2e). A total of 82 of the previously reported TSSs correspond to genes that encode hypothetical proteins where 21 are incorrectly annotated within the CDS. For the remaining 47, we identified TSSs at a different position in 44 the corresponding gene. The three remaining genes in which we failed to identify a TSS are *fliQ*, *fliR*, and *tnp*. Both *fliQ* and *fliR* are predicted to be involved in motility. Motility has been reported to be important for attachment to plant hosts, and *ΔflaABC* mutants were non-motile and are attenuated for virulence (32). However, the role of fliQ and fliR has not been subject to further experimentation (33). The TSS identification algorithm may have not identified a TSS for the flagellar export as a result of overfitting of the optimized parameters or the gene may have low expression in the growth conditions we selected. The *tnp* is predicted to encode a transposase that may have been lost or mobilized and is located within a portion of the pAt plasmid that can be deleted in *A. fabrum* C58 (34). This deletion has been shown to increase expression of virulence genes in *A. fabrum* C58 while also reducing the burden of the plasmid on the host cell (34).

### Genome-wide TSS architecture across the three main lineages of agrobacteria

**Fig 3:**
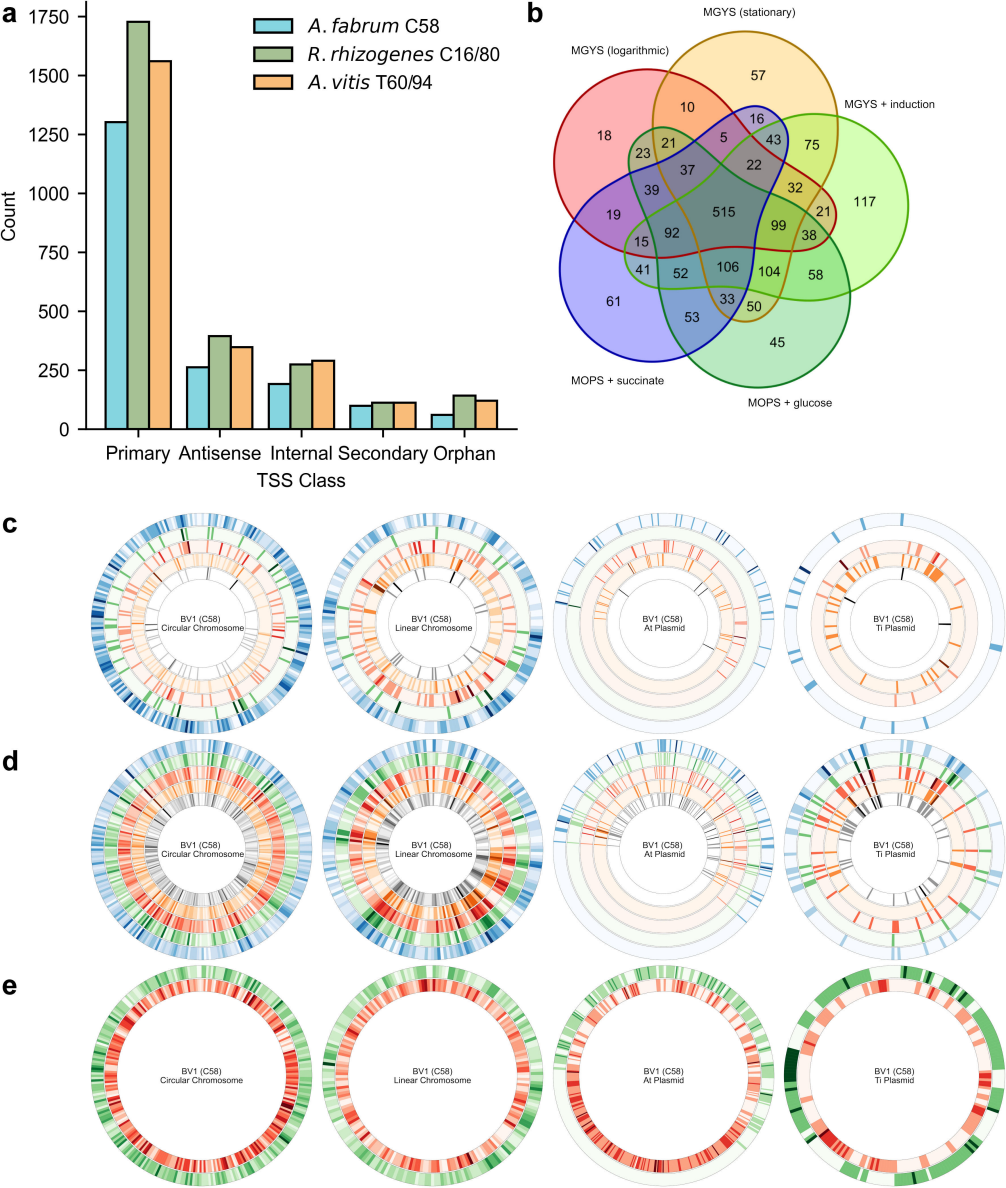
Comprehensive genome-wide TSS identification across replicons, growth conditions, and operons. (**a**) TSS counts by class across *A. fabrum* C58, *R. rhizogenes* C16/80, and *A. vitis* T60/94. (**b**) Overlap of TSS counts by growth conditions for *A. fabrum* C58. (**c**) TSS class by replicons *A. fabrum* C58. The concentric rings show TSS within a 10-kb window for chromosomes and 1 kb for plasmids for primary TSS (blue), secondary TSS (green), internal TSS (red), antisense TSS (orange), and orphan TSS (black). (**d**) TSS by growth condition across replicons for *A. fabrum* C58 for MOPS + glucose (blue), MOPS + succinate (green), MGYS + induction (red), MGYS in logarithmic phase (orange), and MGYS in stationary phase (black). (**e**) Predicted operon coverage for *A. fabrum* C58 with (green) and without (red) an identified primary TSS. There is incomplete coverage of the pAt plasmid due to a common deletion.

To evaluate whether genomic locations of TSSs are conserved across the strains representative of the three main biovars of agrobacteria, we analyzed and compared the distribution of TSS classes. After parameter optimization and validation on *A. fabrum* C58, we performed TSS identification for *R. rhizogenes* C16/80 and *A. vitis* T60/94 (Fig. 3a). Using default parameters, we identified 13,995 TSSs in *R. rhizogenes* C16/80 and 11,792 TSSs in *A. vitis* T60/94 (Table 2). Parameter optimization detected 2,650 TSSs in *R. rhizogenes* C16/80 and 2,432 TSSs in *A. vitis* T60/94 (Table 2). Comparing the overlap of identified TSSs using optimized parameters, 2,503 TSSs (94.5%) in *R. rhizogenes* C16/80 and 2,279 TSSs (93.7%) in *A. vitis* T60/94 are also found in the default parameters data sets (Fig. S3). Of the 6,998 optimized TSSs identified across the three lineages, 6,624 (94.7%) intersect with default parameters. Overall, the relative number of primary, secondary, internal, antisense, and orphan TSSs are similar across the three lineages, with primary TSSs vastly outnumbering other classes after parameter optimization (Fig. 3a). Many of these primary TSS are only expressed in one condition, which might be an indication of condition-dependent transcription (Fig. 3b). Next, we sought to understand positional contributions to gene expression across replicons (Fig. 3c). In addition to the TSS position on replicons, we examined the distribution of condition-dependent TSSs across replicons (Fig. 3d). In *A. fabrum* C58, there were 425 (30.3%), 549 (29.9%) in *R. rhizogenes* C16/80, and 397 (23.7%) in *A. vitis* T60/94 primary and secondary TSSs expressed exclusively in one condition (Fig. S6).

**TABLE 2:** TSS classification across replicons^*a*^

Strain	Replicon	TSS class	TSS count	
			Default parameters	Optimized parameters
*A. fabrum* C58	Circular chromosome	Primary	1,511	823
		Secondary	467	69
		Antisense	684	70
		Internal	1,100	100
		Orphan	93	35
	Linear chromosome	Primary	838	407
		Secondary	275	29
		Antisense	997	76
		Internal	1,484	99
		Orphan	73	19
	At plasmid	Primary	144	54
		Secondary	29	1
		Antisense	152	27
		Internal	190	27
		Orphan	15	4
	Ti plasmid	Primary	70	18
		Secondary	0	0
		Antisense	170	19
		Internal	243	36
		Orphan	45	3

^*a*^ TSS counts for *A. fabrum* C58*, R. rhizogenes* C16/80, and *A. vitis* T60/94 are classified into five categories based on their relative position to the CDS. TSS counts from the default and optimized data sets.

In addition to gene-level TSS identification, our study provides comprehensive coverage of TSSs at the operon level throughout the entire genome. We used an operon prediction pipeline (35) to annotate operons in the genome sequences of *A. fabrum* C58, *R. rhizogenes* C16/80, and *A. vitis* T60/94. In *A. fabrum* C58, we calculated 48.7% coverage of TSSs for all predicted operons, which account for 56.1% of all genes when the pAt deletion described by Morton et al. is taken into consideration (Fig. 3e). Similarly, in *R. rhizogenes* C16/80, we calculated 41.7% coverage of predicted operons which account for 48.6% of genes. In *A. vitis* T60/94, we calculated 53.5% coverage of all predicted operons, which account for 58.8% of genes. Operon coverage maps for *R. rhizogenes* C16/80 and *A. vitis* T60/94 are provided in Fig. S5. A comprehensive summary of TSS counts by bacterial species, replicon, and TSS class in the data sets using default and optimized parameters can be found in Table 2.

### Transcriptional regulation of cell cycle control across agrobacteria

**Fig 4:**
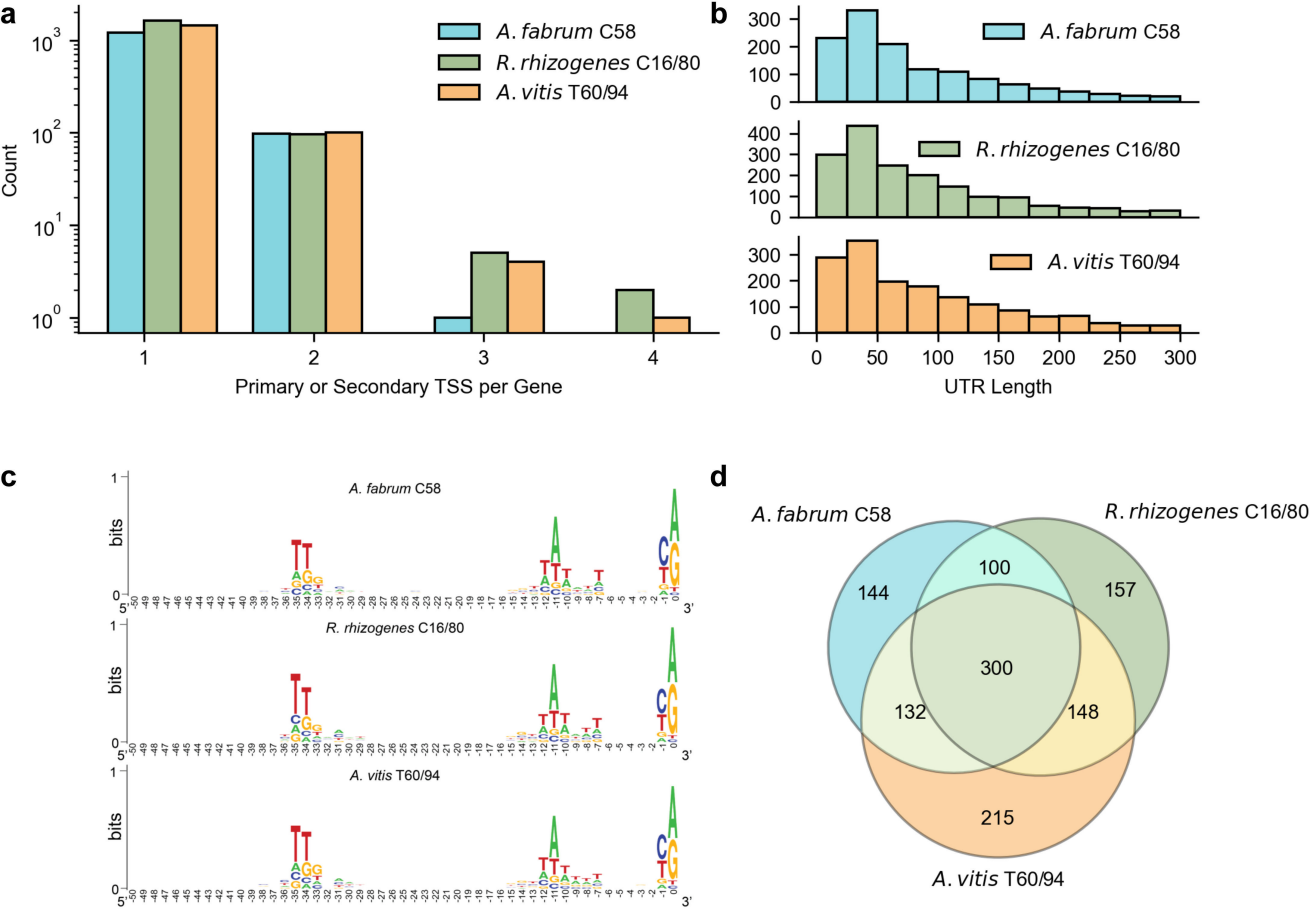
5*'* UTR and promoter structures are largely conserved across agrobacteria. (**a**) Primary and secondary TSS per gene across *A. fabrum* C58, *R. rhizogenes* C16/80, and *A. vitis* T60/94. (**b**) 5*'* UTR length distribution across *A. fabrum* C58, *R. rhizogenes* C16/80, and *A. vitis* T60/94 calculated as the distance between primary TSS and the CDS start. (**c**) Sequence logos of promoters across *A. fabrum* C58, *R. rhizogenes* C16/80, and *A. vitis* T60/94 defined as 50 bases upstream of the TSS where position 0 corresponds to the primary TSS, (**d**) conservation of primary TSS across orthologous clusters.

Alternative regulation programs in specific conditions may be driven by transcription initiation using alternative sigma factors at secondary TSSs. We determined the number of primary and secondary TSSs corresponding to individual genes (Fig. 4a). Most genes have only a primary TSS, although there were instances where one gene had several corresponding secondary TSS. In *A. fabrum* C58, we identified 99 genes with a primary TSS and at least one secondary TSS, and 112 genes for both *R. rhizogenes* C16/80 and *A. vitis* T60/94. In *A. fabrum* C58, *R. rhizogenes* C16/80, and *A. vitis* T60/94, there was a maximum of four TSSs (one primary and three secondary TSSs) for a single gene (Fig. 4a). Across the three lineages, there were a total of 305 genes that were found to have a primary TSS and at least one secondary TSS (Fig. 4a). The identification of primary TSS enables the analysis of the 5*'* untranslated region and promoter structure for each lineage. A total of 4,590 primary TSSs were identified, 92.3% were purines (2,294 A and 1,943 G), and 7.7% were pyrimidines (205 T and 148 C). UTR length appears to be consistent across biovars with a median length of 61 nucleotides (Fig. 4b).

Comparing promoter conservation across species can highlight different or similar transcriptional features and regulation. We compared promoter sequences in the three lineages by extracting the 50 nucleotides upstream from the primary TSS. Sequence logos for promoters of each strain show enrichment of conserved −10 and −35 motifs upstream of detected TSSs, consistent with regions being *bona fide* promoters (Fig. 4c). The promoter structures we identified are consistent with those predicted for Alphaproteobacteria representatives as well as the identified promoter of repABC2 (36, 37). An ortholog clustering analysis recovered a total of 2,362 gene orthologs across lineages with at least one primary TSS and 300 orthologs with primary TSSs in all three agrobacterial genomes (Fig. 4d). Functional enrichment analysis for orthologs with a primary TSS identified in all three lineages shows that promoters driving expression of genes involved in energy production and conversion, post-translational modification, and cell wall biogenesis are highly conserved (Fig. S7). For example, the promoters of cold shock, phosphate starvation, and ribosomal orthologs had high pairwise similarity. We would expect these promoters to be conserved since they are under high selective pressure (38). Orthologous clustering of genes with a primary and at least one secondary TSS expressed exclusively in one condition showed that this subset of TSS is only conserved in the virulence-inducing conditions where 27 orthologs share condition-specific TSSs in at least two species (Fig. S8). Of these 27 genes, we found only one, orthologs of *atu2173*—a methyl-accepting chemotaxis protein—that had condition-specific primary and secondary TSSs conserved across all lineages. Chemotaxis toward plant signals is the first step in the attachment of agrobacteria to plant surfaces, which might explain the presence of this TSS in conditions that simulate the plant environments. Noticeably, of the 17 induction-specific primary and secondary TSSs shared only between *R. rhizogenes* C16/80 and *A. vitis* T60/94, 15 primary and secondary TSSs were upstream of predicted methyl-accepting chemotaxis proteins. The importance of environmental perception under inducing conditions exclusively in *R. rhizogenes* C16/80 is highlighted by 10 of the 29 primary and secondary TSSs as LysR-type transcriptional regulators, which are often associated with metabolite sensing (39). The three remaining genes include two genes annotated as altronate dehydrogenases (orthologs of *atu2822* and *atu3817*) and an ABC transporter (ortholog of *atu4661*) whose homolog in *S. meliloti* was shown to be involved in raffinose transport (40). This suggests that specific sugar metabolism may also be uniquely turned on in *R. rhizogenes* C16/80 under traditional virulence inducing conditions. Of the 39 induction-specific primary and secondary TSSs identified within *A. vitis* T60/94, seven are upstream of genes predicted to encode components of the flagellum, which may explain some of the unique motility (41) and systemic pathology phenotypes (5) observed in *A. vitis* isolates. In addition to flagellar genes, *A. vitis* T60/94 has three unique primary and secondary TSSs upstream of predicted non-heme chloroperoxidases (orthologs of *atu3493*, *atu4778*, and *atu5389*) all located on its second plasmid. As homologs of these genes are also found on both the pSymA and pSymB plasmids of *S. meliloti* (42), this may reflect a conserved defense against halogenated compounds among some *Rhizobium*.

**Fig 5:**
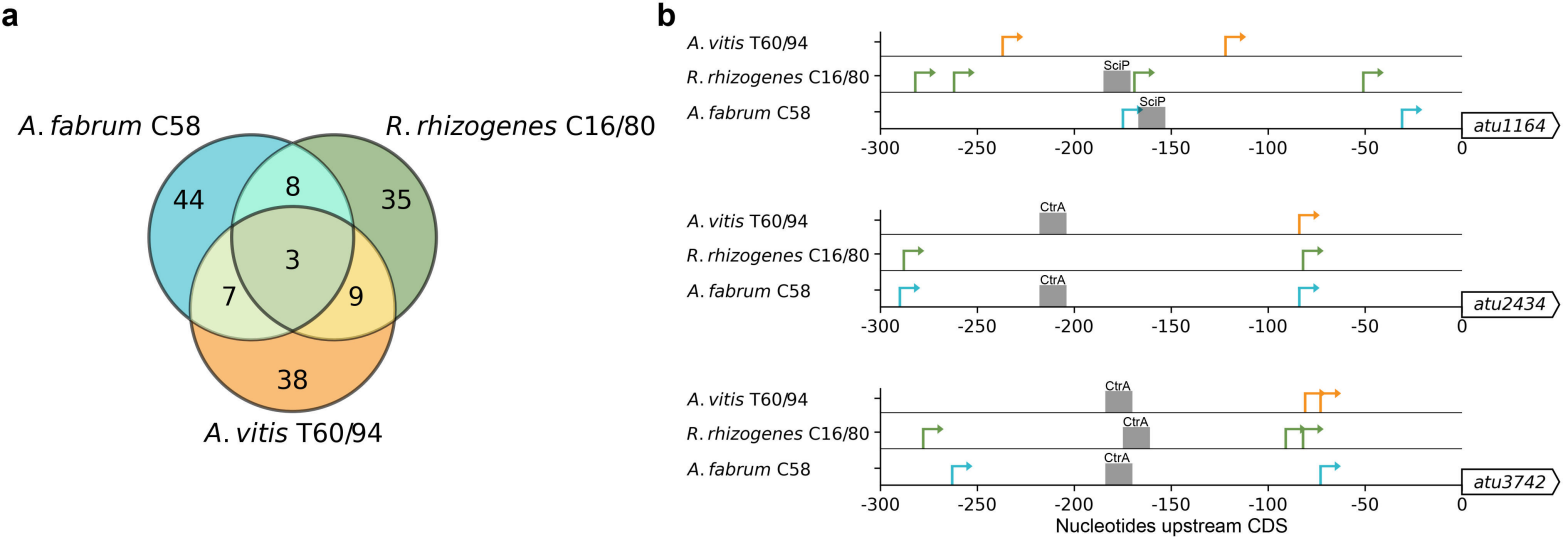
TSS identification uncovers the transcriptional regulation of the cell cycle across agrobacteria. (**a**) Overlap of primary and secondary TSS by ortholog across *A. fabrum* C58, *R. rhizogenes* C16/80, and *A. vitis* T60/94. (**b**) Primary and secondary TSS distribution upstream of CDS orthologs controlled by cell cycle regulators. The gray box indicates the corresponding binding motif for SciP or CtrA across *A. fabrum* C58, *R. rhizogenes* C16/80, and *A. vitis* T60/94.

Ortholog clustering analysis revealed 27 genes in which at least one of its members has one or more secondary TSSs. Of these 27 genes, 10 are predicted to function in the cell cycle, including major cell cycle regulators *ctrA*, *cpdR*, *mraZ*, and *rcdA*, and modifying the cell wall. Previous work in *Caulobacter cresentus* has shown that important cell cycle regulators often had two independently regulated promoters to ensure robust control of cell division (43). All members of three ortholog clusters (represented by *atu1164*, *atu1363*, and *atu3742* of *A. fabrum* C58) have multiple TSSs (Fig. 5a). Two of these genes have known essential cellular functions with *atu1363* encoding proteolytic complex member *clpAS1* (44) and *atu3742* encoding *rcdA* which controls the cell cycle regulator *ctrA* (45). The remaining gene, *atu1164*, is predicted to encode an L,D-transpeptidase shown in *A. fabrum* C58 to localize heterogeneously along the cell membrane and play a role in polar growth strategies (46).

To evaluate whether these genes could be regulated via different regulatory mechanisms across the three lineages, we identified sequence motifs for SciP and CtrA binding sites from *C. cresentus* (43). We mapped these binding sites onto the 10 genes predicted to function in the cell cycle and in modifying the cell wall. We identified possible SciP binding sites upstream of orthologs for *atu1164* and possible CtrA binding sites upstream of *atu2434* and *atu3742* (Fig. 5b). We also propagated the TSS distributions and CtrA binding site predictions to orthologs previously reported to be regulated by CtrA in *A. fabrum* C58 (47, 48) (Fig. S9). Our findings suggest that these binding sites are further upstream relative to their location in *C. cresentus*. However, this is consistent with previous studies in *S. meliloti,* which is more closely related to *A. fabrum* C58*, R. rhizogenes* C16/80, and *A. vitis* T60/94 than *C. cresentus* (49, 50). The distance of primary and secondary TSSs relative to CDS startis plotted for the remaining seven genes with cell cycle and cell wall modifying predicted functions in Fig. S10.

## DISCUSSION

In this study, we identified thousands of TSSs with nucleotide resolution to significantly expand the characterization of promoters and genome-wide transcriptional regulation across the three main lineages of agrobacteria. We extended upon the 356 TSSs associated with CDS previously reported by Wilms et al. for *A. fabrum* C58, representative of BV1, by identifying 1,916 TSSs (338 TSS/Mbp) in our data set. In addition, we completed genomes and phenotyping of BV2 representative *R. rhizogenes* C16/80 and BV3 representative *A. vitis* T60/94 that can enable further research in these less well-studied lineages of agrobacteria. Using their completed genomes, we identified 2,650 TSSs (359 TSS/Mbp) in *R. rhizogenes* C16/80 and 2,432 TSSs in *A. vitis* T60/94. The total number of TSSs identified per organism is consistent with the 1,907 TSSs (1,170 TSS/Mbp) identified in *H. pylori* (16) as well as four *C. jejuni* strains, NCTC11168 with 1,905 TSSs (1,162 TSS/Mbp), 81-176 with 2,003 TSSs (1,178 TSS/Mbp), RM1221 with 2,167 TSSs (1,217 TSS/Mbp), and 81116 with 1,944 TSSs (1,193 TSS/Mbp) (24). These studies used manual curation or validation to optimize the accuracy of their TSS identification. In studies with less optimization or validation, the number of reported TSSs is significantly higher. For example, the 17,001 TSSs (2,541 TSS/Mbp) identified in *S. meliloti* 1021 (19) and 14,868 TSSs (3,204 TSS/Mbp) identified in *E. coli* MG1655 (17) are consistent with the TSS counts in the agrobacterial strains using default parameters. This likely indicates the use of TSS identification methods that are underfitting, thereby resulting in false positives. These results are a reminder that algorithms, especially as they apply toward high-throughput analysis of biological data sets, have limited accuracy without parameter optimization on manually annotated data. This lack of parameter optimization and stringent reproducibility standards in genomics can lead to inaccurate representations and interpretations in understanding transcriptional regulation as well as biological phenomena as a whole.

Full coverage of TSSs for all genes in these organisms will require a better understanding of genome-wide operon induction conditions, especially on plasmids. Genome-wide TSS identification enabled analysis of promoter structure and 5′ UTR features that are consistent with other Alphaproteobacteria representatives. Our TSS identification can also be used toward promoter engineering for synthetic circuits across lineages. These data can contribute to improved identification of transcription factor binding sites and understanding of 5′ UTR contributions to conditional gene expression and regulation in agrobacteria.

In the plant environment, agrobacteria perceive host signals by driving complex transcriptional regulation programs. Some of these natural circuits have been engineered for plant biotechnology purposes, and genome-wide TSS identification of agrobacteria is a crucial step toward mapping global transcriptional changes that occur when the circuits are activated during host infection. In this study, we identify conserved regulation of housekeeper, cell cycle, and cell wall functions. We specifically focus on CtrA- and SciP-mediated transcriptional regulation of agrobacteria promoters and predict their binding sites relative to TSSs in agrobacteria. We identified multiple promoters in *A. fabrum* C58, *R. rhizogenes* C16/80, and *A. vitis* T60/94 upstream of putative cell cycle-regulated genes. These findings are consistent with the differential transcriptional regulation programs found in C. *cresentus* (43) and *S. meliloti* (49, 50). The bacterial cell cycle has largely been studied in Alphaproteobacteria, especially *Caulobacter* species, and the regulation of cell cycle in the close relative of agrobacteria, *S. meliloti*, shows adaptive contributions to host-associated lifestyle (51). Differences in cell cycle regulation of the three main lineages of agrobacteria may contribute to their unique pathologies. Future studies should focus on mapping the complete regulatory networks of cell cycle control by performing binding site analysis based on our TSS data set in these organisms to better understand the evolution of cell cycle control.

Understanding agrobacteria in the context of plant infection can be used toward improving plant biotechnology efforts (52). Our TSS data set enables identification of conserved and divergent transcriptional regulation during virulence-inducing conditions. Expression of motility genes in these conditions suggests that chemotaxis appears to be a hallmark of infection by *R. rhizogenes* C16/80 and *A. vitis* T60/94. Motility genes expressed exclusively in *A. vitis* T60/94 during virulence induction conditions is consistent with previous studies (5, 41) and highlights the importance of chemotaxis during plant infection by *A. vitis* T60/94. Expression of putative sugar transporters in *R. rhizogenes* C16/80 suggests that specific sugar metabolism may also be uniquely turned on during plant infection. We identified numerous LysR-type transcriptional regulators expressed exclusively in *R. rhizogenes* C16/80 that might be important for sensing plant metabolites. Future studies should consider making strains with deletions as well as constitutively expressed LysR-type transcriptional-regulated genes to further understand the contribution of metabolite sensing in disease severity.

In addition to biotechnology applications, the genetic contributions to host range and the unique pathologies across lineages of agrobacteria remain to be elucidated. Understanding the genome-wide transcriptional regulation can facilitate a more complete representation of the evolution of natural genetic circuits. Our completed genomes and optimized TSS provide a step toward completely mapping the transcriptional regulation architecture of agrobacteria.

## MATERIALS AND METHODS

### Strains, media, and culture conditions

*R. rhizogenes* C16/80 and *A. vitis* T60/94 were verified by PCR and Sanger sequencing of *virD2,* following methods previously described (53). *A. fabrum* C58, *R. rhizogenes* C16/80, and *A. vitis* T60/94 were grown in MGYS (10 g/L mannitol, 2.0 g/L L-glutamic acid, 1.0 g/L KH_2_PO_4_, 0.65 g/L K_2_HPO_4_, 0.40 g/L yeast extract, 0.10 g/L NaCl, 0.20 g/L MgSO·7H_2_O, 0.10 g/L CaCl_2_·2H_2_O, 0.010 g/L FeCl_3_·6H_2_O, 0.020 g/L MnSO_4_·H_2_O, 0.020 g/L ZnSO_4_·7H2O, 0.020 g/L CuSO_4_·5H_2_O, 0.0020 g/L H_3_BO_3_, 0.0020 g/L CoCl_2_, 0.020 g/L Na_2_MoO_4_, 0.010 g/L biotin, 0.010 g/L calcium pantothenate, 0.010 g/L thiamine, pH 7.0), 523 (10.0 g/L sucrose, 8.0 g/L casein enzymatic hydrolysate, 4.0 g/L yeast extract, K_2_HPO_4_, MgSO·7H_2_O, pH 7.0), R2A (BD Biosciences, USA), MOPS Minimal Media (32.5 µM CaCl_2_, 0.29 mM K_2_SO_4_, 1.32 mM K_2_HPO_4_, 8 µM FeCl_2_, 40 mM MOPS, 4 mM tricine, 0.01 mM FeSO_4_, 9.52 mM NH_4_Cl, 0.52 mM MgCl_2_, 50 mM NaCl, 0.03 µM (NH_4_)_6_Mo_7_O_24_, 4 µM H_3_BO_3_, 0.3 µM CoCl_2_, 0.1 µM CuSO_4_, 0.8 µM MnCl_2_, and 0.1 µM ZnSO_4_, pH 7.0) supplemented with either 0.1% glucose or 0.1% succinate as a carbon source, and LB Miller medium (BD Biosciences, USA).

### Growth curve measurements

Growth kinetics of agrobacterial strains were carried out as previously described with slight modification (54). Briefly, optical density at 600 nm (OD600) was monitored for 60 hours in a BioTek Synergy 4 plate reader (Agilent Technologies, USA) at 28°C with fast continuous shaking. Cultures were grown in 200 µL volume in 96-well plates for 60 hours in either MGYS, MOPS minimal media with 0.1% glucose as a carbon source, MOPS minimal media with 0.1% succinate as a carbon source, R2A, or LB media (Fig. S1).

### Antibiotic susceptibility testing

Agrobacterial strains were tested for antibiotic susceptibility by performing 11 twofold dilutions of each antibiotic (1,000 ng/mL carbenicillin, 200 ng/mL gentamicin, 1,000 ng/mL kanamycin, 500 ng/mL chloramphenicol, 1,000 ng/mL spectinomycin, 50 ng/mL tetracycline, 1,000 ng/mL hygromycin, and 1,000 ng/mL apramycin) in MGYS across 11 columns of a 96-well plate. The 12th column contained MGYS with no antibiotics. Bacteria were grown in MGYS at 28°C to exponential phase then diluted 50-fold into wells of the 96-well plate. The plate was incubated at 28°C, and OD600 measurements were collected in a BioTek Synergy 4 plate reader (Agilent Technologies, USA) with fast continuous shaking for 48 hours. Antibiotic susceptibility was determined by normalizing the OD600 with a blank MGYS well, then selecting the lowest antibiotic concentration at which the normalized OD600 fell below 50% of the OD600 strain grown in MGYS without antibiotics.

### Genome sequencing and annotation

*R. rhizogenes* C16/80 and *A. vitis* T60/94 genomes were sequenced and assembled as previously described (55). Briefly, sequencing libraries were prepared using the Native Barcoding Kit (Oxford Nanopore Technologies, United Kingdom) and sequenced on an Oxford Nanopore MinION sequencer with SUP basecalling on a MinION flow cell (R9.4.1). For *R. rhizogenes* C16/80, Flye v2.9 with the default options was first used to assemble the long reads, and this assembly was used as input to unicycler with the options “--mode bold --existing_long_read_assembly” (56). For *A. vitis* T60/94, Unicycler v.0.4.7 with the option “--mode normal” was used to hybrid assemble previously generated Illumina short reads and Nanopore long reads (2, 57). Bandage v.0.8.1 was used to visualize assembly completeness and structure (58). Bakta v.1.5.0 was used to annotate genome assemblies (59). NCBI assembly GCA_000092025.1 was used for *A. fabrum* C58. Updated genome assemblies are available on NCBI with assembly IDs GCA_013321855.2 (*R. rhizogenes* C16/80) and GCA_013337045.2 (*A. vitis* T60/94).

### dRNA-seq library preparation

To map TSSs to genome sequences, we developed a custom dRNA-seq protocol based on previous methods that use conditional treatment with TEX (20). Agrobacterial strains were struck out on MGYS agar plates and grown overnight at 28°C. Three biological replicates were selected from each plate and inoculated individually into separate tubes with 5 mL of MGYS then grown overnight at 28°C. The following morning, each culture was spun down and washed twice in PBS buffer then inoculated at a 50-fold dilution into MGYS, MOPS + glucose, and MOPS + succinate, respectively. After 18 hours, OD600 measurements were taken every hour. The final OD600 for each biological replicate is shown in Fig. S2. Cell pellets were spun down and frozen at 80°C. Nucleic acid isolation was carried out using bead beating and Qiagen RNeasy Kits (Qiagen, Germany), followed by DNA depletion using the DNA-free Kit (Invitrogen, USA). RNA was cleaned using Zymo RNA Clean & Concentrator 5 Kit (Zymo Research, USA). Ribosomal RNA was depleted using Illumina Ribo-Zero Kit (Illumina, USA). Samples were split into terminator 5′-phosphate-dependent exonuclease (Lucigen, USA) treated and untreated groups then cleaned using RNA Clean & Concentrator 5. All samples were treated with RppH (New England Biolabs, USA) to remove pyrophosphates from the 5′ end of triphosphate RNA then cleaned using Zymo RNA Clean & Concentrator 5. A conserved RNA adaptor (5′ ACACUCUUUCCCUACACGACGCUCUUCCGAUCU 3′) was synthesized (Integrated DNA Technologies, USA), treated with FastAP Alkaline Phosphatase (Thermo Fisher Scientific, USA), then ligated to the RNA samples using T4 RNA Ligase 1 (New England Biolabs, USA). Residual RNA adapters were removed by AMPure XP (Beckman Coulter, USA) bead cleanup. The ligated RNA was used for first-strand cDNA synthesis using SuperScript IV First-Strand Synthesis System (Invitrogen, USA) and a DNA adaptor (5′ GTGACTGGAGTTCAGACGTGTGCTCTTCCGATCTNNNNNN 3′) that adds a conserved 3′ sequence to the cDNA library. Residual DNA adapters were removed by AMPure XP bead cleanup. Sequencing barcodes were added by qPCR using SsoAdvanced Universal SYBR Green Supermix (Bio-Rad Laboratories, USA) to minimize PCR-based bias. Residual primers were removed by AMPure XP bead cleanup. Library extraction metrics are shown in Fig. S2. The UC Berkeley QB3 sequencing facility normalized and sequenced libraries on an Illumina NovaSeq 6000 with 100-bp paired-end reads. Sequencing reads from dRNA-seq are publicly available on SRA Database under BioProject accession PRJNA921868.

### TSS data analysis

Illumina adapter sequences were removed from sequencing reads using trimmomatic (60) then filtered based on the presence of the 5*'* adapter using seqtk (61) to an average of 15,083,319 reads per sample after adapter trimming. Segemehl (62) was used to align filtered reads to their corresponding reference genome. We observed high alignment between dRNA-seq reads and the reference genomes with an average of 97.44% overall alignment rate per library. A custom Perl script was used to convert alignment files into WIG files that are required by the TSS identification algorithm in the ANNOgesic package (29). TSSs from the linear chromosome of *A. fabrum* C58 for parameter optimization were manually curated as previously described (16). The ANNOgesic docker image was used to create project directories and perform TSS optimization and prediction.

### Ortholog clustering

We performed ortholog clustering of protein sequences across the three BV genomes using PIRATE v.1.0.4 (63). We selected a 50% amino acid sequence similarity threshold to compare the conservation and divergence of orthologs as previously described (21). TSS data sets from ANNOgesic and ortholog clustering from PIRATE were analyzed in Python v.3.10. All code is publicly available on GitHub (https://github.com/shih-lab/agrobacteria_TSS).

## SUPPLEMENTAL MATERIAL

File S1 msystems.00333-23-s0001.xlsx Agrobacteria TSS.

File S2 msystems.00333-23-s0002.xlsx Operon predictions.

File S3 msystems.00333-23-s0003.xlsx Orthologous clustering.

Supplemental Material msystems.00333-23-s0004.docx Supplemental figures and table.

ASM does not own the copyrights to Supplemental Material that may be linked to, or accessed through, an article. The authors have granted ASM a non-exclusive, world-wide license to publish the Supplemental Material files. Please contact the corresponding author directly for reuse.
