# Supplementary material for: Transcriptome architecture of the three main lineages of agrobacteria
Source: mSystems. 2023 Jul 21;8(4):e00333-23. doi: 10.1128/msystems.00333-23 (PMC10469942; doi:10.1128/msystems.00333-23)
Supplement: Supplemental Material — Supplemental figures and table. [file msystems.00333-23-s0004.docx]

**Supplementary Information**

**Figure S1. Effects of growth conditions on the growth kinetics of agrobacterial strains** Growth curves for **(a)** *A. fabrum* C58*,* **(b)** *R. rhizogenes* C16/80, and **(c)** *A. vitis* T60/94 in MGYS, MOPS+glucose, MOPS+succinate, R2A, and LB media.

**
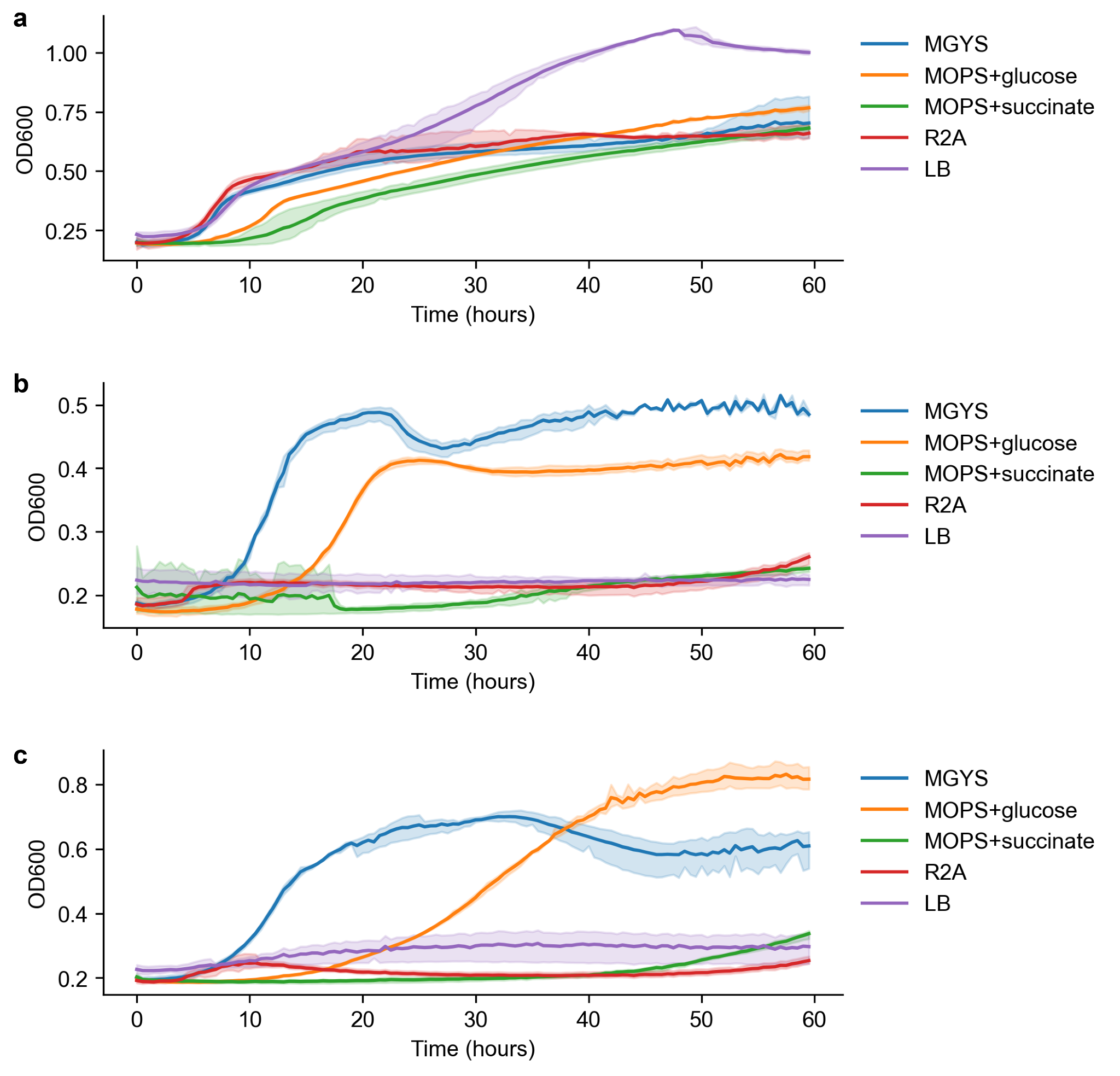
**

**Figure S2. Sequencing library preparation metrics.** OD600 at sample collection, RNA concentration at extraction, DNase treatment, and RNA cleanup by growth condition for **(a)** *A. fabrum* C58*,* **(b)** *R. rhizogenes* C16/80, and **(c)** *A. vitis* T60/94.

**
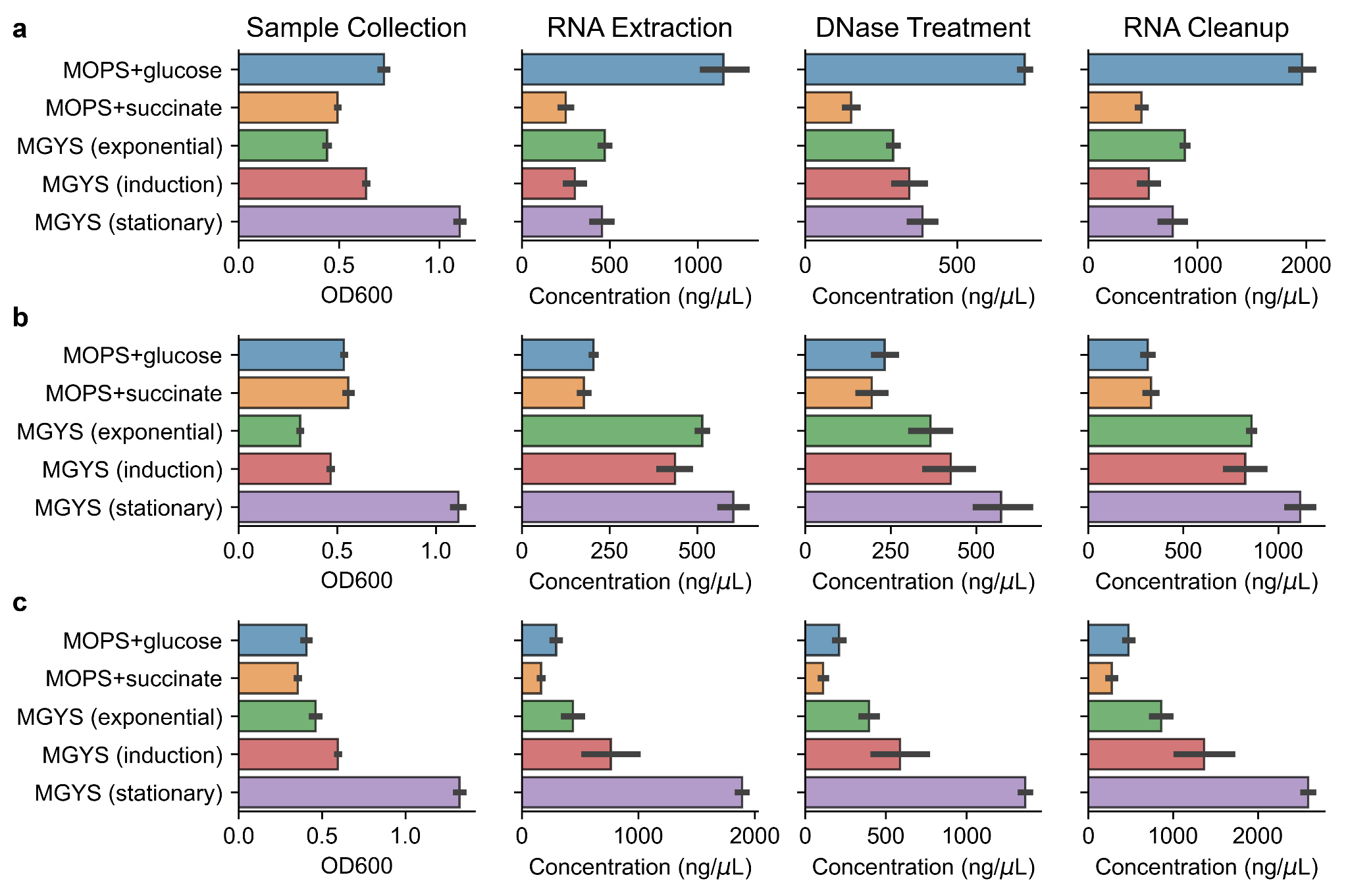
**

**Figure S3. Optimized TSS identification in BV2 and BV3.** TSS counts using default and optimized parameters as well as overlaps of TSS counts between the default and optimized datasets for **(a)** *R. rhizogenes* C16/80 and **(b)** *A. vitis* T60/94.

**
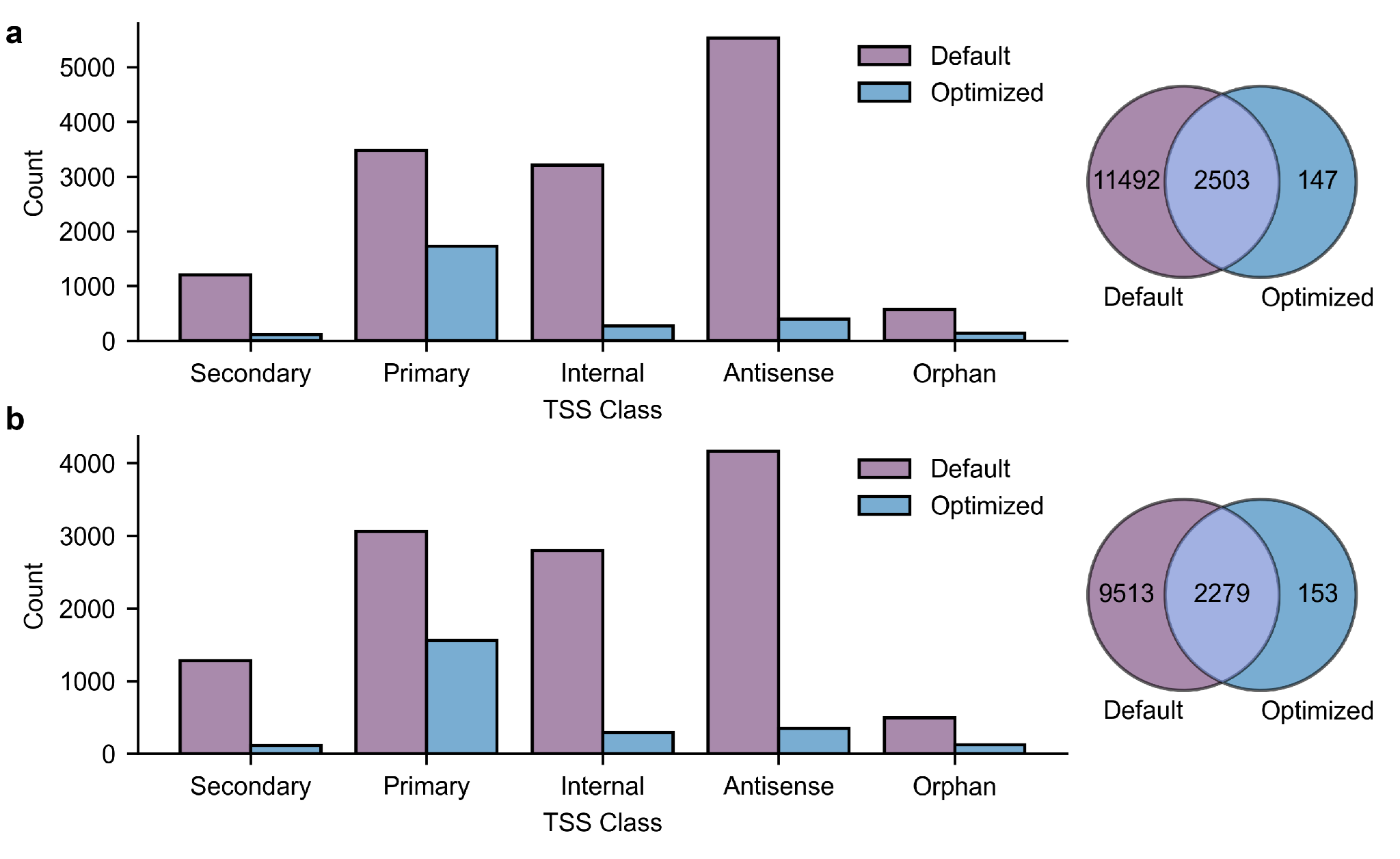
**

**Figure S4. Relative TSS counts by class across replicons.** TSS counts normalized by replicon size for **(a)** *A. fabrum* C58*,* **(b)** *R. rhizogenes* C16/80, and **(c)** *A. vitis* T60/94.

**
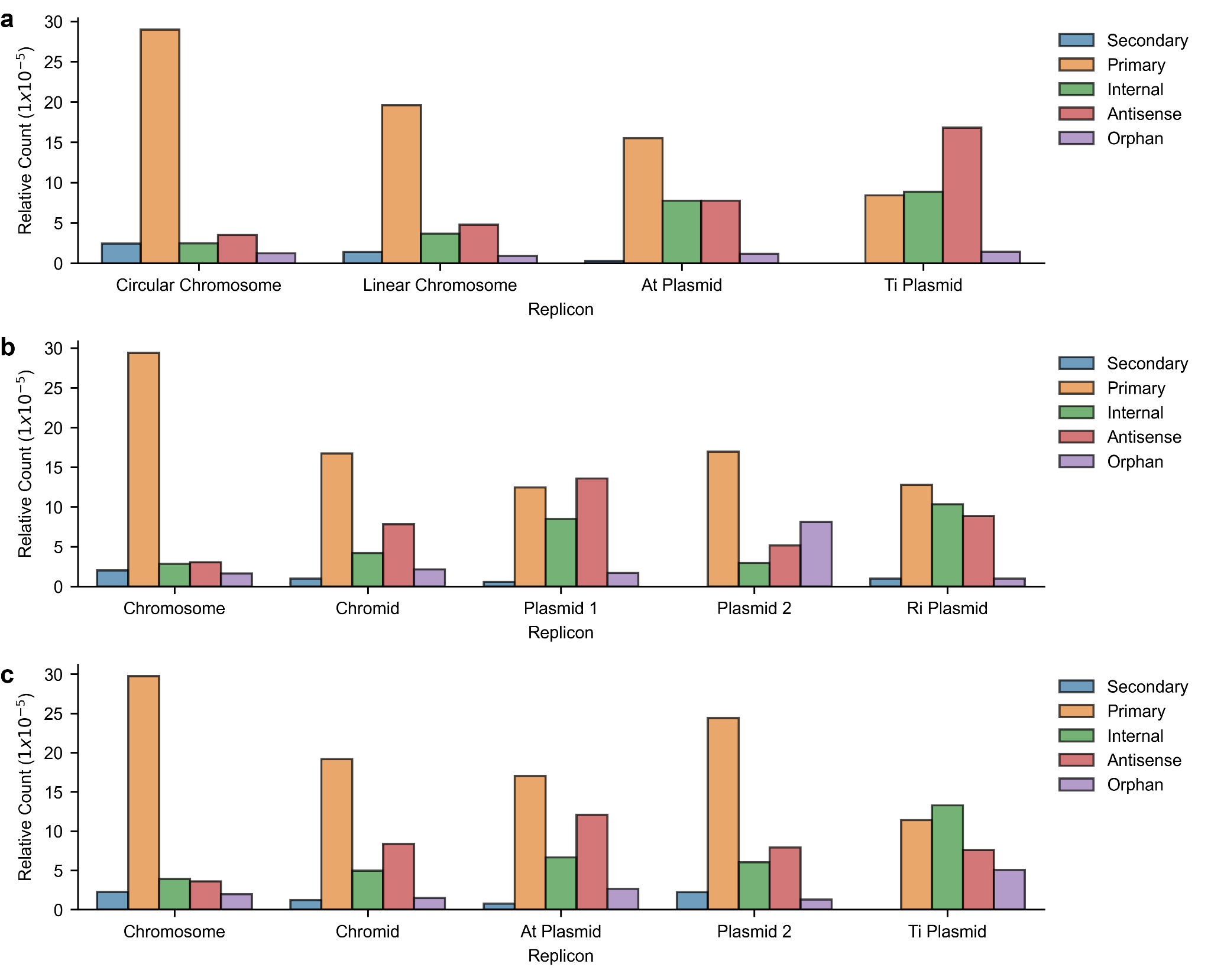
**

**Figure S5. Genome-wide TSS identification across replicons, growth conditions, and operons for BV2 and BV3.** TSS by class, growth conditions, and operon coverage across replicons for **(a-d)** *R. rhizogenes* C16/80 and **(e-h)** *A. vitis* T60/94. The largest contig for plasmid 2 of C16/80 is shown.


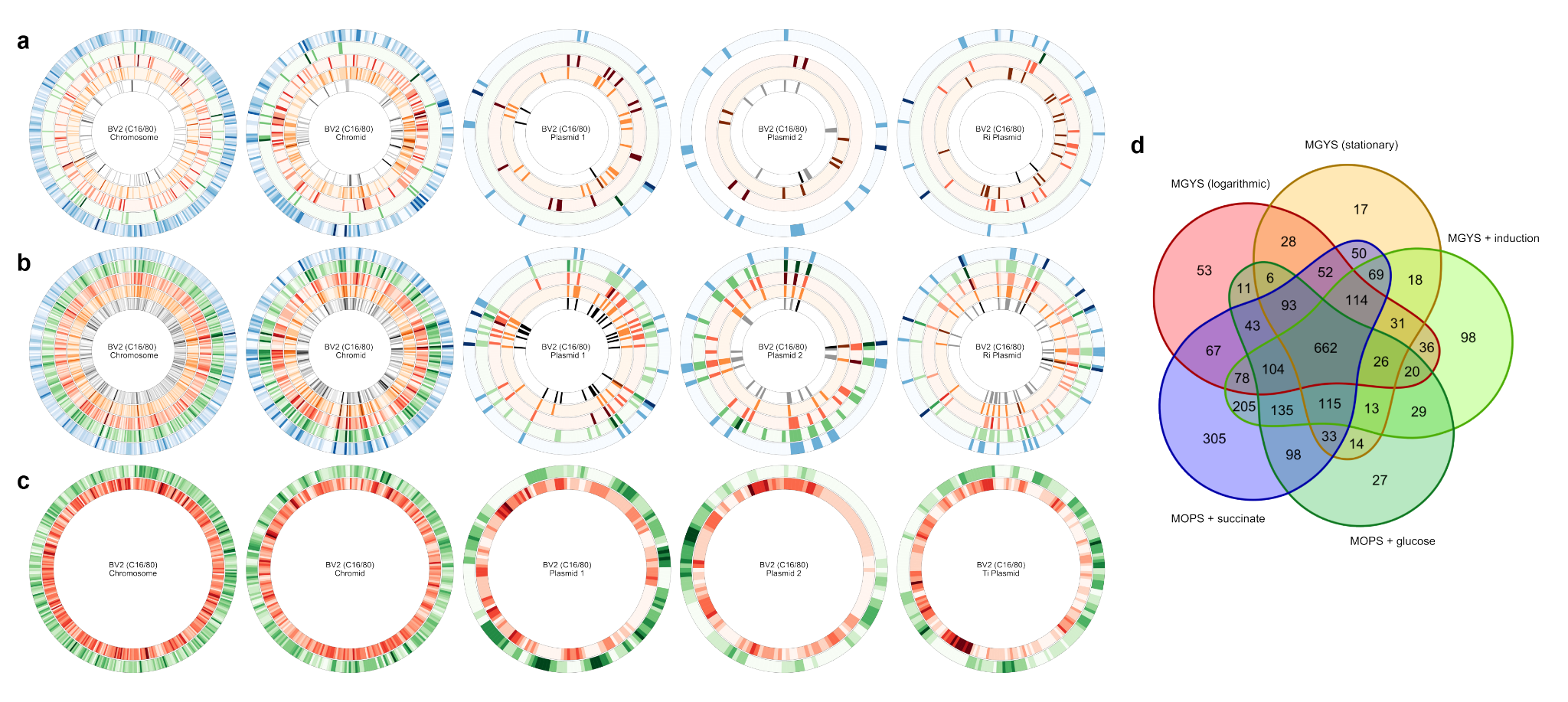


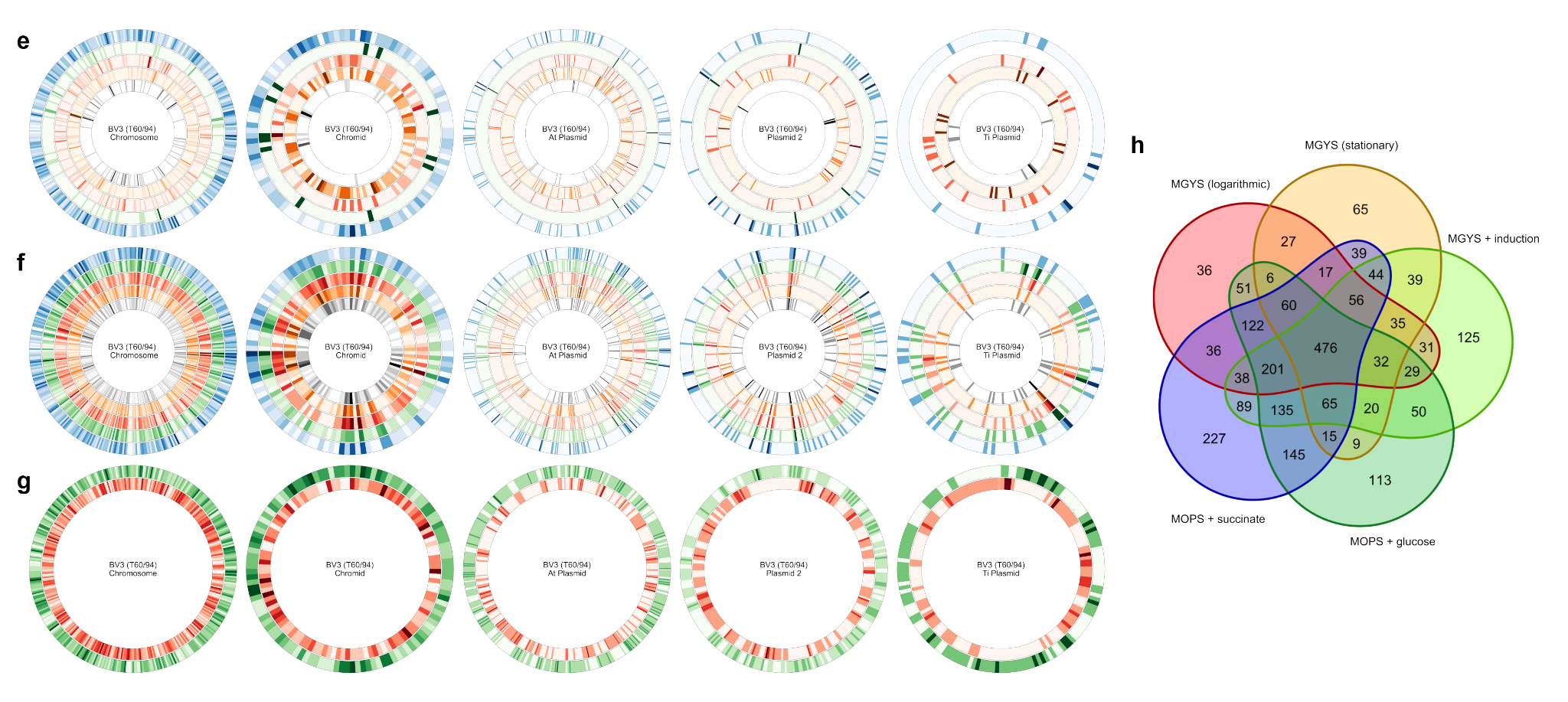


**Figure S6. Relative primary and secondary TSS counts by condition across lineages.** TSS counts normalized by overall TSS for *A. fabrum* C58*, R. rhizogenes* C16/80, and *A. vitis* T60/94.

**
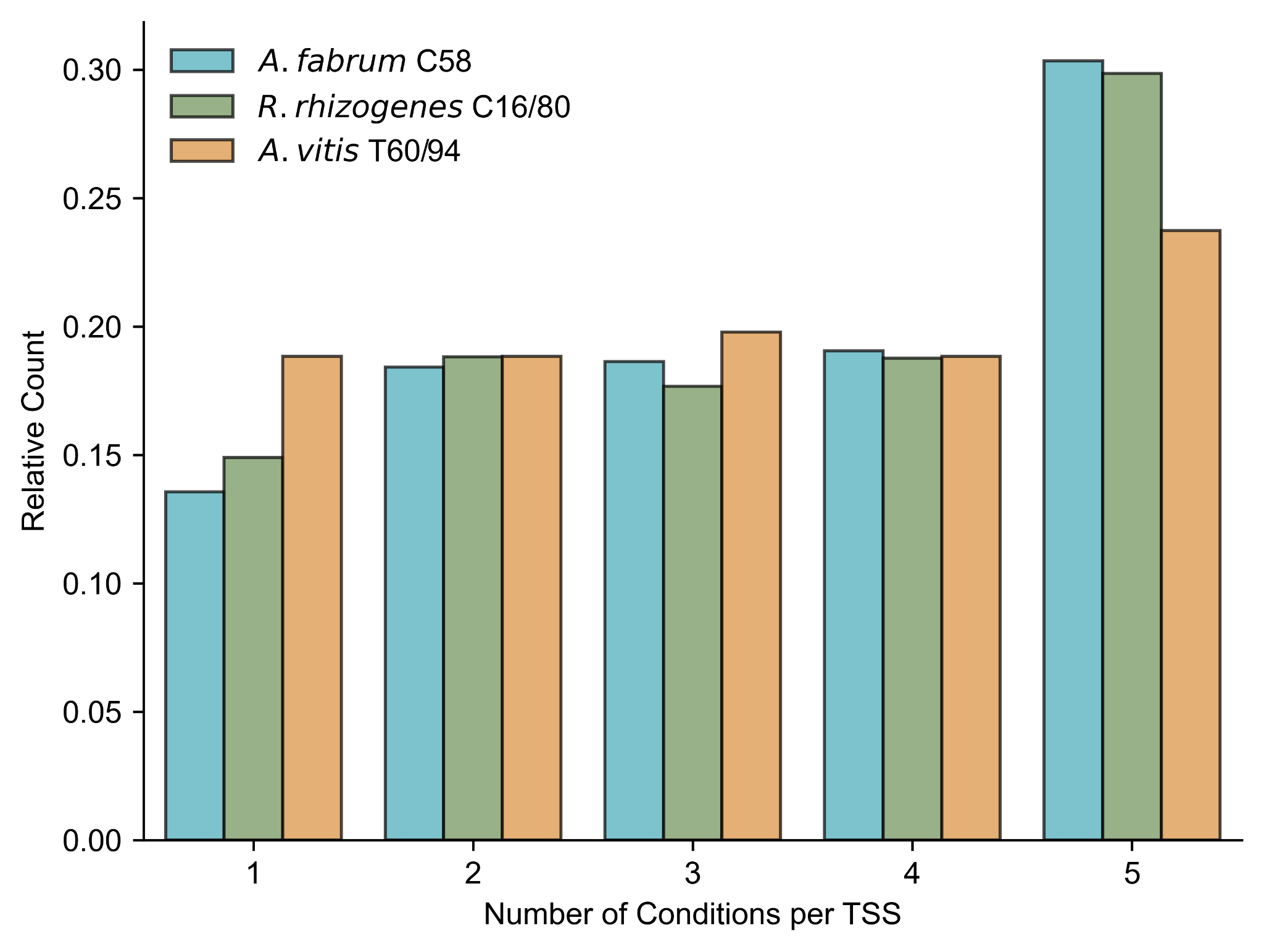
**

**Figure S7. Functional enrichment for promoters of orthologs with conserved primary TSS across biovars. a)** Pairwise alignment scores for promoters of orthologs with primary TSS in *A. fabrum* C58*,* *R. rhizogenes* C16/80, and *A. vitis* T60/94. Orthologs are grouped as one standard deviation above or below the mean alignment score as conserved (blue) and divergent (red). **b)** Enrichment analysis of the conserved subset relative to the divergent subset based on COG category.

**
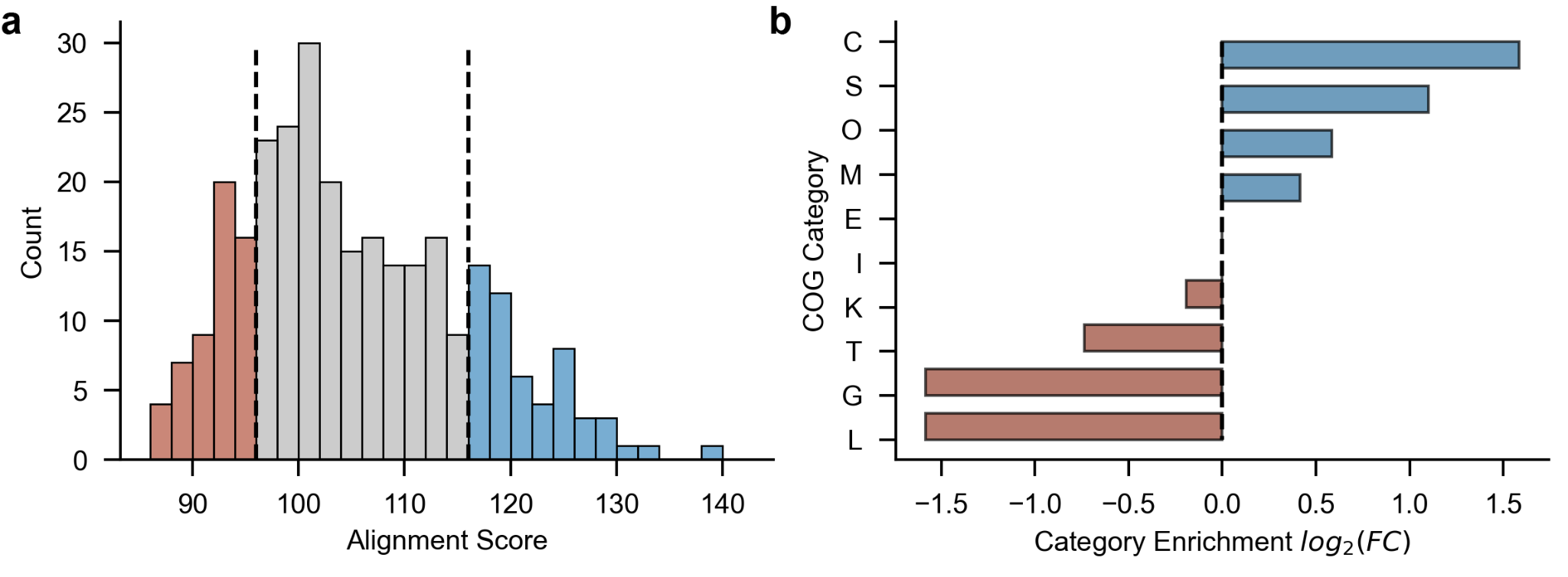
**

| **COG Category** | **Description** |
| --- | --- |
| C | Energy production and conversion |
| E | Amino Acid metabolism and transport |
| G | Carbohydrate metabolism and transport |
| I | Lipid metabolism |
| K | Transcription |
| L | Replication and repair |
| M | Cell wall/membrane/envelope biogenesis |
| O | Post-translational modification, protein turnover, chaperone functions |
| S | Function Unknown |
| T | Signal Transduction |

**Figure S8. Primary and secondary TSS expressed exclusively in one condition across biovars.** Overlaps of primary and secondary TSS expressed exclusively in, **(a)** MGYS + induction, **(b)** MGYS in logarithmic phase, **(c)** MGYS in exponential phase, **(d)** MOPS+succinate, **(e)** MOPS+glucose for *A. fabrum* C58*,* *R. rhizogenes* C16/80, and *A. vitis* T60/94.

**
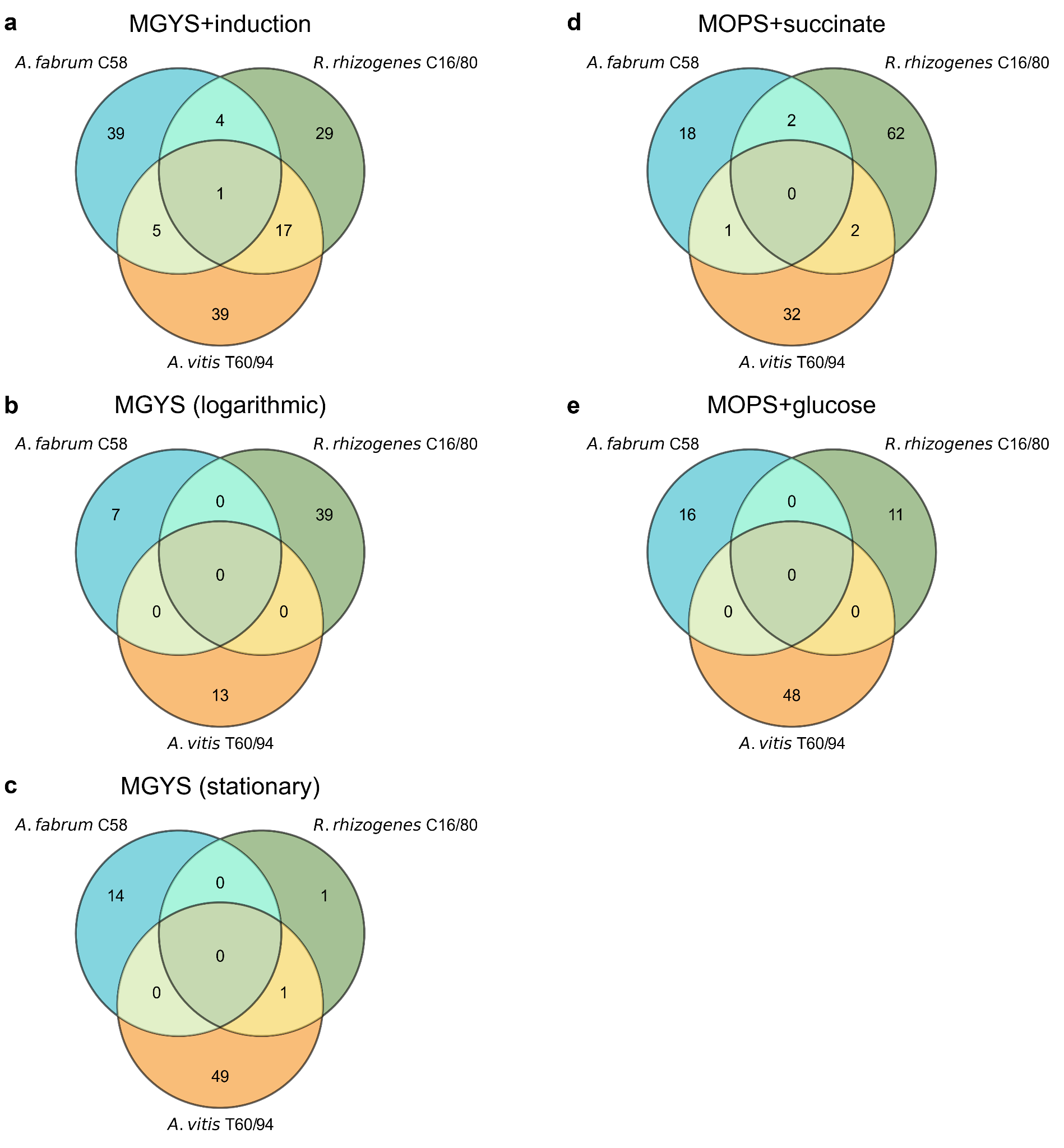
**

**Figure S9. TSS distribution across cell cycle regulation genes as well as predicted CtrA-controlled orthologs.** Ortholog clustering for *A. fabrum* C58 (blue), *R. rhizogenes* C16/80 (green), *A. vitis* T60/94 (orange) indicating the position of TSS upstream of the CDS with the annotation corresponding to the *A. fabrum* C58 ortholog. Gray boxes indicate a predicted CtrA binding motif.

**
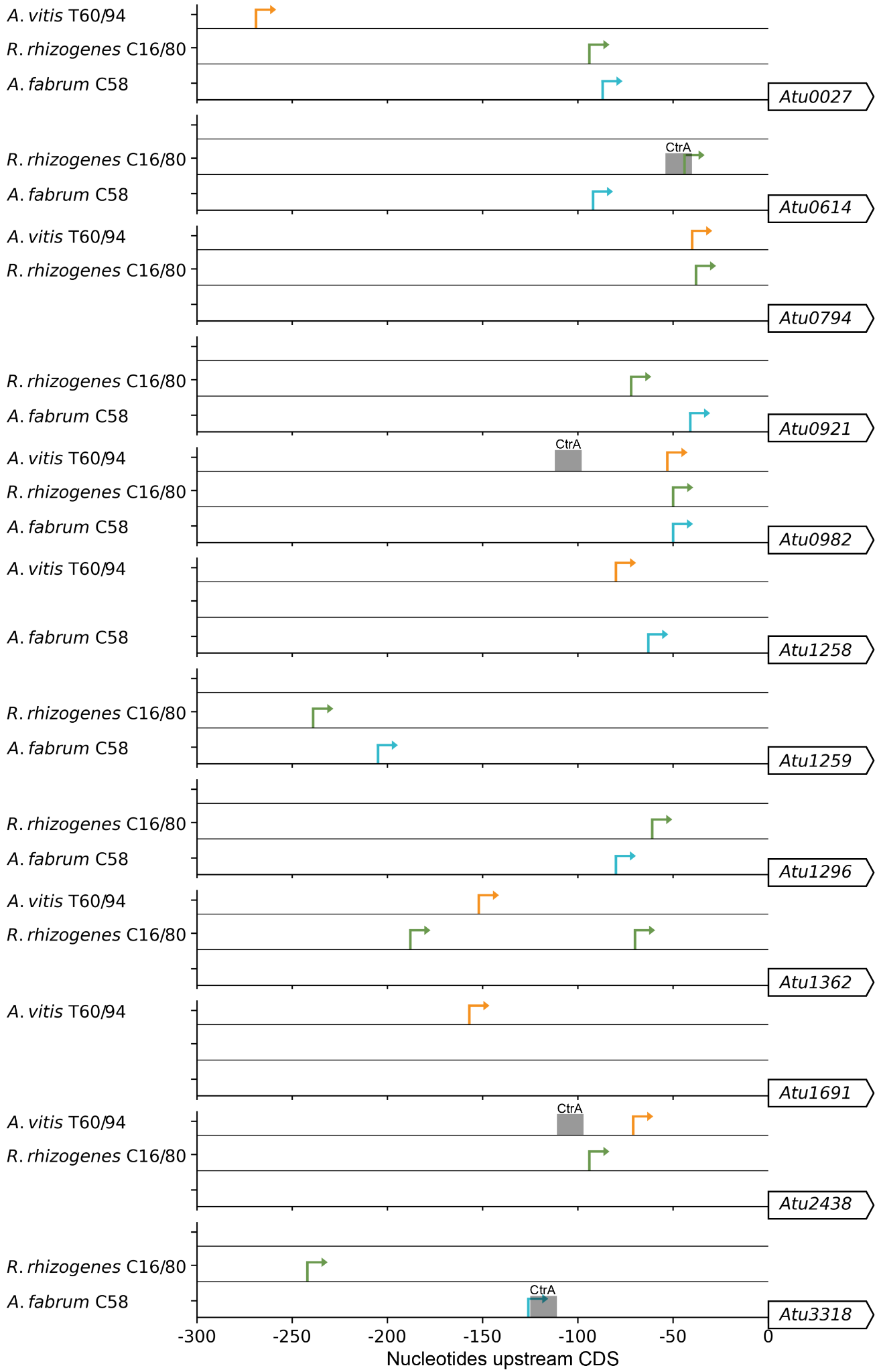
**

**Figure S10. Primary and secondary TSS distribution across conserved cell cycle and cell wall modifying orthologs with at least one secondary TSS.** Ortholog clustering for *A. fabrum* C58 (blue), *R. rhizogenes* C16/80 (green), *A. vitis* T60/94 (orange) indicating the position of TSS upstream of the CDS with the annotation corresponding to the *A. fabrum* C58 ortholog.

**
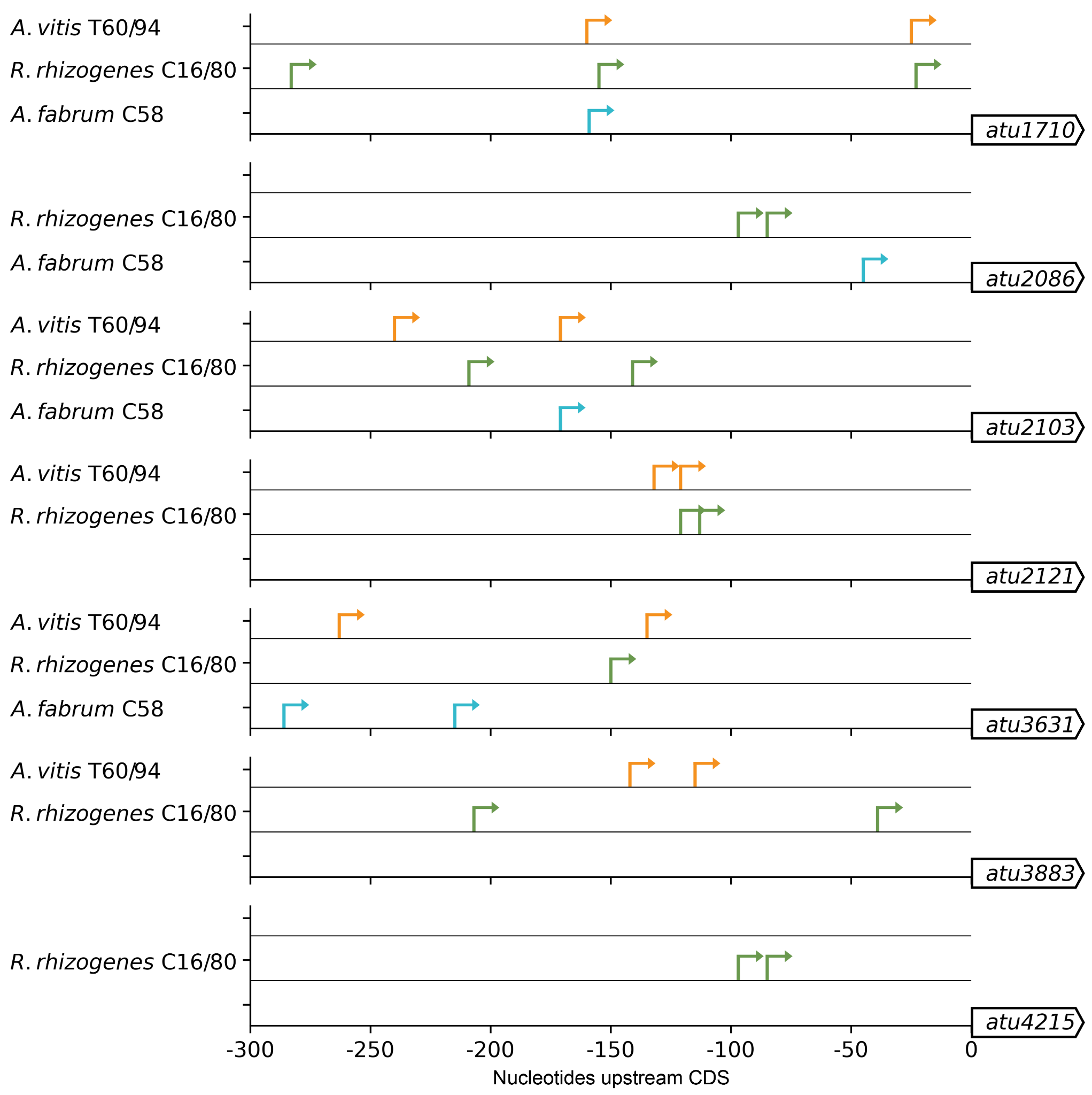
**

**Table S1. Antibiotic susceptibility testing.** Minimal inhibitory concentration (MIC) for *Agrobacterium fabrum* C58*, Rhizobium rhizogenes* C16/80, and *Allorhizobium vitis* T60/94 in carbenicillin (Carb), gentamicin (Gent), kanamycin (Kan), chloramphenicol (Cam), spectinomycin (Spec), tetracycline (Tet), hygromycin (Hyg), and apramycin (Apr).

|  | **Antibiotic MIC (ug/mL)** | | | | | | | |
| --- | --- | --- | --- | --- | --- | --- | --- | --- |
| **Strain** | **Carb** | **Gent** | **Kan** | **Cam** | **Spec** | **Tet** | **Hyg** | **Apr** |
| *A. fabrum C58* | 15 | 1 | 5 | 5 | 5 | 0.5 | 5 | 1 |
| *R. rhizogenes* C16/80 | 15 | 1 | 5 | 5 | 50 | 0.5 | 5 | 1 |
| *A. vitis* T60/94 | 250 | 1 | 5 | 5 | 1 | 0.5 | 5 | 1 |
